# The conservation of allelic DNA methylation and its relationship with imprinting in maize

**DOI:** 10.1093/jxb/erad440

**Published:** 2023-11-03

**Authors:** Xiaomei Dong, Haishan Luo, Jiabin Yao, Qingfeng Guo, Shuai Yu, Yanye Ruan, Fenghai Li, Weiwei Jin, Dexuan Meng

**Affiliations:** College of Bioscience and Biotechnology, Shenyang Agricultural University, Shenyang 110866, Liaoning, China; Shenyang City Key Laboratory of Maize Genomic Selection Breeding, Shenyang 110866, Liaoning, China; College of Agronomy, Shenyang Agricultural University, Shenyang 110866, Liaoning, China; College of Agronomy, Shenyang Agricultural University, Shenyang 110866, Liaoning, China; College of Agronomy, Shenyang Agricultural University, Shenyang 110866, Liaoning, China; College of Bioscience and Biotechnology, Shenyang Agricultural University, Shenyang 110866, Liaoning, China; Shenyang City Key Laboratory of Maize Genomic Selection Breeding, Shenyang 110866, Liaoning, China; College of Bioscience and Biotechnology, Shenyang Agricultural University, Shenyang 110866, Liaoning, China; Shenyang City Key Laboratory of Maize Genomic Selection Breeding, Shenyang 110866, Liaoning, China; College of Agronomy, Shenyang Agricultural University, Shenyang 110866, Liaoning, China; State Key Laboratory of Plant Physiology and Biochemistry, National Maize Improvement Center, Key Laboratory of Crop Heterosis and Utilization, the Ministry of Education, College of Agronomy and Biotechnology, China Agricultural University, Beijing 100193, China; Department of Agronomy, College of Agriculture & Resources and Environmental Sciences, Tianjin Agricultural University, Tianjin 300392, China; College of Agronomy, Shenyang Agricultural University, Shenyang 110866, Liaoning, China; New Zealand Institute for Plant and Food Research Limited, New Zealand

**Keywords:** DNA methylation, imprinted genes, interspecific conservation, intraspecific conservation, kernel development, maize, *Zea mays*

## Abstract

Genomic imprinting refers to allele-specific expression of genes depending on parental origin, and it is regulated by epigenetic modifications. Intraspecific allelic variation for imprinting has been detected; however, the intraspecific genome-wide allelic epigenetic variation in maize and its correlation with imprinting variants remain unclear. Here, three reciprocal hybrids were generated by crossing *Zea mays* inbred lines CAU5, B73, and Mo17 in order to examine the intraspecific conservation of the imprinted genes in the kernel. The majority of imprinted genes exhibited intraspecific conservation, and these genes also exhibited interspecific conservation (rice, sorghum, and Arabidopsis) and were enriched in some specific pathways. By comparing intraspecific allelic DNA methylation in the endosperm, we found that nearly 15% of DNA methylation existed as allelic variants. The intraspecific whole-genome correlation between DNA methylation and imprinted genes indicated that DNA methylation variants play an important role in imprinting variants. Disruption of two conserved imprinted genes using CRISPR/Cas9 editing resulted in a smaller kernel phenotype. Our results shed light on the intraspecific correlation of DNA methylation variants and variation for imprinting in maize, and show that imprinted genes play an important role in kernel development.

## Teaser

Reciprocal crosses in maize indicate that the majority of imprinted genes show intraspecific and interspecific conservation, and two conserved imprinted genes are shown to be important for kernel development

## Introduction

Genomic imprinting, which is observed in flowering plants and in mammals, refers to a biased expression of alleles that depends on the parent of origin (Kermicle, 1970). Maize seed development initiates from the process of double-fertilization, in which two sperms fuse with an egg cell and a central cell to produce an embryo and endosperm, respectively (Dumas *et al.*, 1993; Chaudhury *et al.*, 2001). Imprinted genes in maize have been found primarily in the endosperm (Waters *et al.*, 2013; Zhang *et al.*, 2014), the function of which is to provide nutrients for the developing and germinating embryo. Imprinted genes have been identified in maize in early immature embryos (Meng *et al.*, 2018).

DNA methylation is a heritable epigenetic mark and it can affect gene transcription and thus influence development. Genomic approaches over the last decade have revealed extensive variation in intraspecific natural DNA methylation (Schmitz *et al.*, 2013; Kawakatsu *et al.*, 2016; Guo *et al.*, 2023). In maize, more than 25% of cytosines in the genome are methylated (Xu *et al.*, 2020). Whole-genome bisulfite sequencing (WGBS) in populations of modern maize, landrace varieties, and the wild ancestor teosinte have found that methylation variations exist widely and possibly contribute to adaptive and phenotypic variations. In both mammals and plants, DNA methylation plays an important role in the epigenetic regulation of genomic imprinting. Genome-wide allele-specific patterns of DNA methylation have been reported in several plant species (Ibarra *et al.*, 2012; Rodrigues *et al.*, 2013; Zhang *et al.*, 2014). Imprinted genes are significantly correlated with differentially methylated regions (DMRs) according to the parent of origin (pDMRs) (Monk *et al.*, 2019); however, research in this area remains limited in maize kernels.

At the intraspecies level, most imprinted genes in plants exhibit conserved imprinting (Waters *et al.*, 2013; Zhang *et al.*, 2016; Yang *et al.*, 2020), but more than 10% exhibit variation in allelic imprinting. In particular, the epiallelic variation of imprinted genes might underlie the variation in seed development phenotypes. For example, *HOMEDOMAIN GLABROUS3* (*HDG3*) in Arabidopsis is a paternally expressed gene (PEG) in the endosperm in Cvi × Col crosses, but is biallelically expressed in the endosperm in Col × Cvi crosses (Pignatta *et al.*, 2014). Loss of *HDG3* imprinting is associated with late endosperm cellularization and changes in seed weight. In addition, variation in DNA methylation contributes to variation in *HDG3* imprinting in Arabidopsis (Pignatta *et al.*, 2018). Hence, a genome-wide survey of the relationship between epigenetic variation and allelic imprinting in maize is important for a better understanding of the relationship between epigenetic variation and phenotypic variation.

Imprinted genes have been shown to participate in several development processes in seeds, including morphogenesis, dormancy, and post-zygotic reproductive isolation (Lu *et al.*, 2013; Piskurewicz *et al.*, 2016; Lafon-Placette *et al.*, 2018; Martinez *et al.*, 2018; Zhu *et al.*, 2018; Iwasaki *et al.*, 2019). For example, *Meg1* (*maternally expressed gene 1*) is a maternal imprinting gene in maize, and *Meg1*-RNAi plants show a phenotype with smaller kernels (Costa *et al.*, 2012). In addition, maize mutants for the male parent-specific imprinting gene *de18*, which has been found to play a critical role in endosperm development, exhibit a large number of aborted kernel phenotypes in mature ears (Bernardi *et al.*, 2012; Hatorangan *et al.*, 2016). However, the number of imprinted genes with known functions or mutant phenotypes is still limited in plants, even though a large number of imprinted genes have been identified using transcriptomics.

In this study, we selected three maize inbred lines, B73, Mo17, and CAU5, for analysis of intraspecifically conserved imprinted genes in maize kernels. The three lines were each used as the male and female parents in reciprocal crosses between them in order to analyse the transcriptome data for the embryos and endosperms at 11 days after pollination (DAP). Among the 599 genes detected in three sets of reciprocal crosses, the majority were interspecifically conserved imprinted genes, and 77 of the genes were also found to be conserved and imprinted in rice, sorghum, and Arabidopsis. We then compared the differentially methylated regions that were dependent on the parent of origin (pDMRs) of the non-conserved genes identified in the reciprocal crosses, and we also examined the relationship between imprinting variation and DNA methylation. Finally, we determined that mutation of two conserved genes affected the kernel size. Our results contribute to understanding the mechanism of action of these imprinted genes in the process of maize kernel development, improve our knowledge of the molecular regulation network of kernel development, and lay a theoretical foundation for increasing maize production.

## Materials and methods

### Tissue collection

The maize (*Zea mays*) inducer line CAU5 and two normal lines, B73 and Mo17, were grown at the experimental station of Shenyang Agriculture University in Shenyang, Liaoning, China. The ears and tassels of the three lines were bagged with kraft paper on the day prior to pollination. The next day, each bag from a tassel was patted to collect the pollen from one parent, and this was then used to pollinate the ear of the other parent, as detailed in Table 1. The ears from each of the crossed were collected after 11 d. Embryo and endosperm tissues were collected by manual dissection.

**Table 1.** List of crosses used in the study

Abbreviation	Cross
BB	Self-cross of B73
MM	Self-cross of Mo17
CC	Self-cross of CAU5
BM	B73 × Mo17
MB	Mo17 × B73
BC	B73 × CAU5
CB	CAU5 × B73
MC	Mo17 × CAU5
CM	CAU5 × Mo17

Given that haploids may be produced in the F_1_ crosses of BC and MC (i.e. when the inducer line was the male parent), the ploidy of each embryo that we extracted was detected by both genome-wide Maize6H-60K SNP sequencing and by using sequencing of 13 KASP markers at chromosome 7 (for primers see Supplementary Table S1). Haploid embryo parts were defined by all the 13 KASP markers and more than 90% of the Maize6H-60K SNPs corresponding to the B73 or Mo17 SNPs in the BC or MC crosses; otherwise, they were defined as diploid embryo parts. Two haploid embryos were separated for RNA-sequencing and verification, and some of the diploid embryos and triploid endosperm from at least three ears in each cross were also prepared for RNA-seq.

### Library construction for RNA-seq and MethylC-seq

In each replicate and the two haploid embryos, RNA samples were isolated using a Quick RNA Isolation Kit (Huayueyang Biotechnology, Beijing). Library construction and sequencing were performed according to the Illumina instructions. Total RNA was used as the input material for the preparation of RNA samples. Sequencing libraries were generated using a NEB Next^®^ Ultra TM RNA Library Prep Kit for Illumina^®^. The construction of the mRNA library and the high-throughput sequencing were performed using the Illumina NovaSeq 6000 platform, and 150 bp paired-end reads were generated. For each replicate ~3 Gb of data were obtained and used in the subsequent analysis. As the transcriptome size of maize is nearly 60 Mb, the sequence depth was ~50×, which was sufficient for our analysis.

Genomic DNA degradation and contamination were checked by agarose gels. DNA purity was checked using a Nano Photometer^®^ spectrophotometer (Implen, CA, USA). DNA concentration was measured using a Qubit^®^ DNA Assay Kit and a Qubit^®^ 2.0 Fluorometer (Life Technologies). Positive control DNAs were added to the DNA samples, which were then broken into 200–400 bp fragments using a Covaris S220 ultrasonicator. The DNA fragments were then treated with bisulfite (Accel-NGS Methyl-seq DNA Library Kit for Illumina, Swift Biosciences), after which adapter ligation, size selection, and PCR amplification steps were performed on the fragments. The library quality was assessed using an Agilent Bioanalyzer 2100 system. Pair-end sequencing of the samples was performed on the Illumina platform.

### Read-mapping, gene expression analysis, and SNP-calling

Clean reads were first aligned to the B73 reference genome (v.4) using HISAT2 with default parameters (Kim *et al.*, 2019). Normalized gene expression values (reads per kilobase of transcript per million fragments mapped, FPKM) were estimated using the Cufflinks software (v.2.2.1) (Trapnell *et al.*, 2012). Normalized data of log_2_(RPKM value + 1) were used to calculate the correlation coefficient.

Resequencing data of B73, Mo17, and CAU5 were downloaded from NCBI (SRR12415217, SRR12415218, SRR3124079). Clean reads were aligned using BWA with default parameters (Li and Durbin, 2009), and SNP-calling was performed using bcftools with default parameters (Li *et al.*, 2009). Finally, we identified 1 669 940 SNPs covering 22 213 genes in the BC/CB cross, 1 588 429 SNPs covering 20 258 genes in the MC/CM cross, and 1 299 229 SNPs covering 19 666 genes in the BM/MB cross to distinguish parental alleles.

### Measurement of allelic expression and identification of imprinted genes

To avoid bias, SNP sites were converted to Mo17 or CAU5 nucleotides to obtain the SNP-substituted genome. Clean reads from three biological replicates of each sample were mapped to the two parent genomes using HISAT2 with default parameters (Kim *et al.*, 2019). Only unique mapped reads were retained. SAM files were converted to BAM files using Samtools (Li *et al.*, 2009). Three replicates from each sample were combined to identify the imprinted genes. After combining the mapping results, the read counts of annotated genes were summarized using Samtools mpileup. Based on the SNP information, we also divided the reads aligned at the SNP site from maternal or paternal alleles using Samtools mpileup. If the summed read counts of annotated genes at all SNP sites was ≥20, then the genes were selected for further analysis in the endosperm. To improve the accuracy of identifying imprinted genes in the embryo, we required the read counts of annotated genes at all SNP sites to be ≥20 in each of the three individual replicates. Genes were subjected to χ^2^ tests to detect the deviation of the parental expression ratio of the SNP locus from 1:1 (embryo) or 2:1 (endosperm). Imprinted maternally expressed genes (MEGs) and paternally expressed genes (PEGs) were identified in the embryo if significant allelic bias (χ^2^ < 0.05) was detected and if >80% of transcripts were derived from the maternal/paternal allele. For the endosperm, imprinted genes were also identified with a significant allelic bias (χ^2^ < 0.05) and with >80% of the transcripts from the maternal allele for MEGs, but with >50% of the transcripts being from the paternal allele for PEGs.

### Gene Ontology enrichment analysis

Gene Ontology (GO) term enrichment analysis for the identified genes was performed using AgriGO v2.0 (Tian *et al.*, 2017). GO terms within the categories ‘cell component’, ‘molecular function’, and ‘biological process’ were identified that showed significant (*P*<0.05) enrichment within at least one cross.

### Pipeline for MethylC-seq analysis

MethylC-seq reads were generated using the same workflow as previously described by Zhang *et al.* (2014). First, low-quality reads were filtered using SolexaQA (Cox *et al.*, 2010) and the remaining reads were mapped to the B73 genome using Bismark (Krueger and Andrews, 2011). The bulk methylation of the endosperm was calculated by the ratio of Cs/(Cs+Ts) from all CG, CHG, and CHH sites. The SNPs were then used to separate allele-specific MethylC sequence reads from the hybrid endosperm. Only sites with at least five reads were used in subsequent analysis. The same criteria were used to identify differentially methylated regions (DMRs) that were dependent on the parent of origin (pDMRs) for the CG and CHG sites (CG_pDMR and CHG_pDMR, respectively), as described by Zhang *et al.* (2014). First, a sliding-window approach with a 200 bp window and 20 bp steps was adopted throughout the genome. Only windows containing more than five CG/CHG sites supported with at least five reads were kept. Second, the statistical significance of the allelic methylation bias in each window was assessed using Fisher’s exact test, and the resulting *P*-values were converted to *Q*-values. Finally, the pDMRs were identified according to the following criteria: FDR<0.01; difference in methylation level between the two alleles >30%; and the hypermethylated alleles had methylation levels >40% in the context of CG. The candidate pDMRs were then further filtered using a smaller window size of 50 bp, and pDMRs within 200 bp were merged.

### Validation of imprinted genes

We randomly tested the status of four SNPs in two imprinted genes detected in our study (*Zm00001d037209* and *Zm00001d022443*) using a PCR-sequencing method. Each gene fragment was amplified using different primers from six cDNA samples from embryos at 11 DAP: BB, MM, CC, BM and MB, or BC and CB, or MC and CM. The primer information is given in .

### Genetic transformation of maize

Two genetic transformation constructs were prepared for two conserved genes, *Zm00001d019342* and *Zm00001d004401*. For the CRISPR/Cas9-edited construct of *Zm00001d019342* (encoding a variant of methylation 104, denoted here as *Zmvim104*), a 19 bp sequence from the first exon was selected as guide RNA (gRNA) and introduced into the pBUE411 vector as previously described (Xing *et al.*, 2014). The construct was introduced into the KN5585 maize receptor line by *Agrobacterium*-mediated transformation (Ishida *et al.*, 2007). Using PCR amplification and sequencing, two independent transgenic lines with a frame-shift in the coding sequence were obtained (T_0_) and self-pollinated twice to generate homozygous progenies (T_2_), which included one line with a 7 bp deletion in the gRNA targeted region (named as *zmvim104-C1*) and another line with a 16 bp deletion (named as *zmvim104-C2*) (for primers, see Supplementary Table S2).

For the *Zm00001d004401* gene (encoding a germin-like protein2, denoted here as *Zmglp2*), a 19 bp sequence from the second exon was selected as the gRNA, and was introduced into the pBUE411 vector as previously described (Xing *et al.*, 2014). The construct was introduced into the maize receptor line KN5585 through *Agrobacterium*-mediated transformation (Ishida *et al.*, 2007). Using PCR amplification and sequencing, two independent transgenic lines with frame-shift in the coding sequence were obtained (T_0_) and self-pollinated twice to generate homozygous progenies (T_2_), which included a 1 bp insertion in the gRNA targeted region (named as *zmglp2-C1*) and a 20 bp deletion (named as *zmglp2-C2*) (Supplementary Table S2).

### Subcellular localization

The subcellular localizations of the Zmvim104 and the Zmglp2 proteins was predicted using DeepLoc (https://services.healthtech.dtu.dk/service.php?DeepLoc-1.0). For determination of the subcellular localization of Zmvim104, we generated the construct *p35S::Zmvim104-GFP* using the full-length CDS of *Zmvim104* without the stop codon. Fragments were amplified by PCR from cDNA prepared from the RNA of immature endosperm (16 DAP) of B73 using the GFP-Zmvim104-F/R primers (Supplementary Table S2). The PCR products were cloned into the KpnI and XbaI sites of pCambia1300-GFP to create GFP-fusion proteins. P2300-35S-H2B-mCherry-RFP was used as the nucleus marker.

For the determination of the subcellular localization of Zmglp2, two constructs were generated: *p35S::Zmglp2-GFP* using the full-length CDS of *Zmglp2* without the stop codon, and the truncated fusion protein *Zmglp2-GFP* (∆Zmglp2-GFP) with the signal peptide sequence removed. The full-length and truncated CDSs of *Zmglp2* were PCR-amplified from cDNA prepared from immature kernels (18 DAP) of B73 using the primers GFP-ZMGLP2-F/R and GFP-tru-ZMGLP2-F/R (Supplementary Table S2). The PCR products were cloned into the KpnI and XbaI sites of pCambia1300-GFP to create fusion proteins with GFP.

The *Zmvim104* and *Zmglp2* plasmids were each transformed into leaf epidermal of tobacco (*Nicotiana benthamiana*) cells as previously described (Zuo *et al.*, 2019). GFP and RFP signals were detected at the 488 nm and 532 nm laser lines, respectively, under an Olympus FV1000 laser scanning microscope.

## Results

### Identification of imprinted genes in maize embryo and endosperm tissues

We performed RNA-seq analysis to identify imprinted genes using immature (11 DAP) embryo and endosperm tissues from the reciprocal crosses among the CAU5, B73 and Mo17 lines (Table 1). An average of 14 M clean reads from each biological replicate were aligned to the reference genome (Supplementary Table S3), and the correlations of the three biological replicate samples in each combination of each tissue were greater than 0.96 (Supplementary Table S4; Supplementary Fig. S1).

**Fig. 1.**
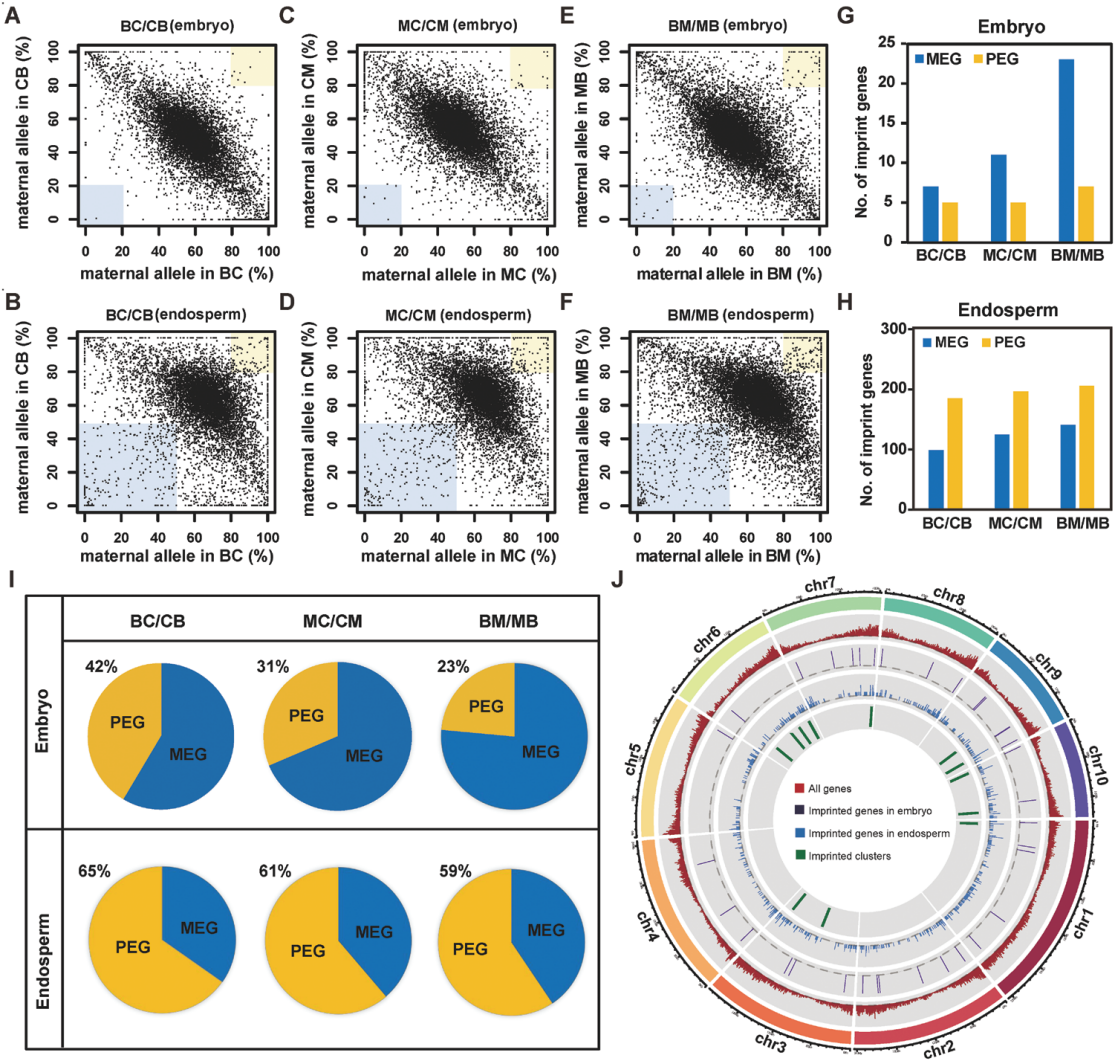
Imprinted genes detected in the embryo and endosperm of reciprocal crosses of three maize inbred lines, B73, CAU5, and Mo17 (Table 1). (A–F) Scatterplots showing the relative transcriptional output of the maternal and paternal alleles of each gene with more than 20 allelic reads in the embryo (A–C) and endosperm (D–F). The yellow and blue shaded areas indicate MEGs (upper right) or PEGs (lower left). (G, H) The numbers of imprinted genes identified in (G) the embryo and (H) the endosperm. MEG, maternally expressed gene; PEG, paternally expressed gene. (I) The proportions of MEGs and PEGs in the imprinted genes identified in the embryo and endosperm of the three reciprocal hybrids. (J) Chromosomal distribution of the imprinted genes.

To identify imprinted genes, we created scatter-plots to visualize the relative transcriptional output of the maternal and paternal alleles of each gene with more than 20 allelic reads (Fig. 1A–F). As shown in Fig. 1G, H, in the BC/CB crosses there were 12 imprinted genes detected in the embryos (seven MEGs and five PEGs) and 282 in the endosperm (98 MEGs and 184 PEGs); in the MC/CM crosses there were 16 in the embryos (11 MEGs and 5 PEGs) and 319 in the endosperm (124 MEGs and 195 PEGs); and in the BM/MB crosses there were 30 in the embryos (23 MEGs and seven PEGs) and 345 in the endosperm (140 MEGs and 205 PEGs). Thus, overall there were more MEGs than PEGs in embryos, while the endosperm contained more PEGs than MEGs (Fig. 1I). Next, we examined the chromosome locations of the imprinted genes that were detected in the three reciprocal crosses (Fig. 1J; Supplementary Table S5). We scanned the genome for candidate clusters containing at least two adjacent imprinted transcripts within a region of 1 Mb, and found that 25 imprinted genes fell into 12 clusters in the endosperm (Supplementary Table S6). Except for one cluster that included both MEGs and PEGs, most imprinted genes within a cluster showed the same parental preference.

To verify the precision of the expression data from RNA-seq, we randomly tested the status of four SNPs in two imprinted genes (*Zm00001d037209* and *Zm00001d022443*). Two SNPs at *Zm00001d037209* were predominantly expressed by maternal alleles in the reciprocal crosses at each SNP site (Supplementary Fig. S2) and two SNPs at *Zm00001d022443* were predominantly expressed by paternal alleles in the reciprocal crosses at each SNP site. These results were consistent with the mRNA-seq data.

### Comparison of the intraspecific imprinting status of genes

**Fig. 2.**
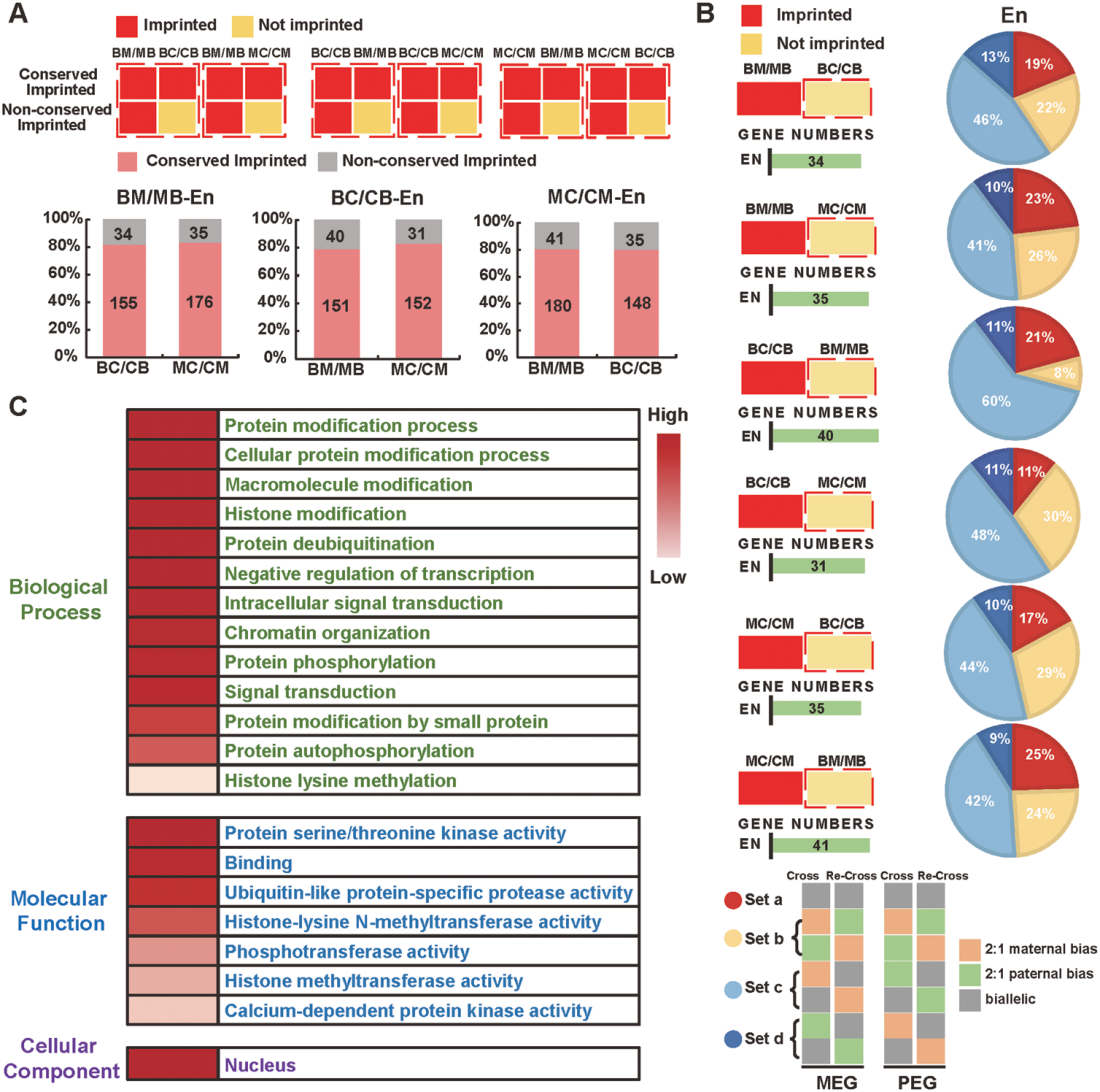
Conservation analysis of the imprinted genes detected in the endosperm (En) of reciprocal crosses of three maize inbred lines, B73, CAU5, and Mo17 (Table 1). (A) Classification and proportions of the conserved and non-conserved imprinted genes in the three reciprocal hybrids. (B) Detailed analysis of the non-conserved genes detected in the three reciprocal hybrids (see also Supplementary Table S5). ‘Set a’ indicates that the gene status was not imprinted in both the cross and re-cross; ‘Set b’ represents genes showing the opposite imprint status in both the cross and re-cross; ‘Set c’ represents genes that show same imprinting status in the cross or in the re-cross; and ‘Set d’ represents genes that show the opposite imprinting direction in the cross or in the re-cross. ‘Cross’ represents BM, BC or MC. ‘Re-cross’ represents MB, CB, or MC. The gene number represents the sum of the four sets a–d. (C) GO analysis of conserved genes. GO terms that showed significant (*P*<0.05) enrichment within at least one cross are shown. The non-conserved genes did not show significant enrichment in any of the pathways.

Due to the limited number of imprinted genes in the embryo, we mainly focused on the intraspecific conservation for imprinted genes in the endosperm. As shown in Fig. 2A, the conserved imprinted genes were those that were imprinted simultaneously in both of the reciprocal hybrids in each of the crosses, while the non-conserved imprinted genes were imprinted in only one of the crosses. Most of the imprinted genes in the endosperm were intraspecifically conserved imprinted genes in maize. For example, among the 345 imprinted genes identified in BM/MB, a total of 189 could be allelically determined in BC/CB and 211 in MC/CM, with 155 (82.0%) and 176 (83.4%) being conserved imprinted genes in BC/CB and MC/CM, respectively. The allelic variation of the non-conserved imprinted genes was then further investigated. As shown in Fig. 2B, the majority of non-conserved imprinted genes were allele-specific imprinted genes, indicating that allelic variation was primarily from allele-specific imprinting; that is, genes will only be maternally/paternally biased when a particular inbred line is the male or female parent.

GO analysis was performed to study the potential functions of the conserved and non-conserved imprinted genes in maize development (Fig. 2C). Conserved imprinted genes were enriched in many pathways in the biological process category, including ‘signal transduction’ and ‘protein modification process’. In the molecular function category, conserved genes were highly enriched in ‘binding’ and ‘protein serine/threonine kinase activity’. In contrast, the non-conserved genes did not show significant enrichment in any of the pathways.

### Comparison of allelic DNA methylation in the endosperm of BM/MB and MC/CM crosses

**Fig. 3.**
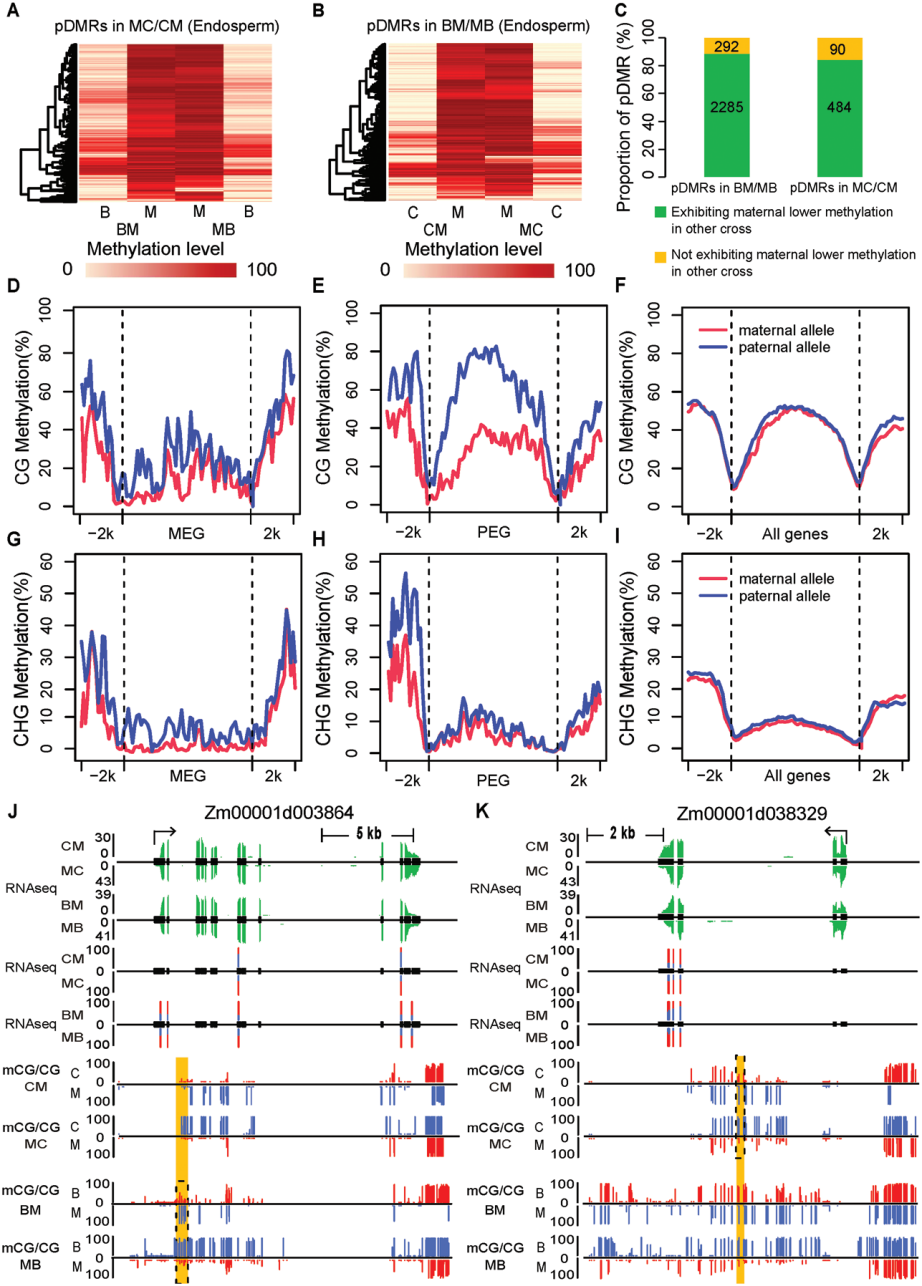
Comparison of allele DNA methylation levels and expression between MC/CM and BM/MB endosperm (see Table 1). (A) Heatmap of methylation levels between alleles of the B73 and Mo17 reciprocal crosses at differentially methylated regions (DMRs) that were dependent on the parent of origin (pDMRs) for the CG sites (CG_pDMRs) identified in MC/CM. (B) Heatmap of CG methylation levels between alleles of CAU5 and Mo17 reciprocal crosses at the CG_pDMRs identified in BM/MB. (C) Comparison of allelic methylation at CG_pDMR identified in MC/CM and BM/MB endosperm. A total of 2285 CG_pDMRs identified in BM/MB endosperm exhibited maternal lower methylation in MC/CM endosperm; 484 CG_pDMRs identified in MC/CM endosperm exhibited maternal lower methylation in BM/MB endosperm; 292 CG_pDMRs identified in BM/MB endosperm did not exhibit maternal lower methylation in MC/CM endosperm; and 90 CG_pDMRs identified in MC/CM endosperm did not exhibit maternal lower methylation in BM/MB endosperm. (D–I) Differential DNA methylation between the two parental alleles for maternally expressed genes (MEGs) and paternally expressed genes (PEGs). The gene body was split into 60 bins, and 2 kb up- and downstream regions were split into 20 bins. (J, K) Integrated profiles of allele-specific gene expression and DNA methylation levels in BM/MB and MC/CM endosperm for the genes *Zm00001d003864* (PEG in BM/MB but non-imprinted in MC/CM) and *Zm00001d038329* (PEG in MC/CM but non-imprinted in BM/MB). The expression levels of genes according to RNA-seq are shown in green, and the percentages of allelic reads for specific SNP sites are shown with red lines for the paternal allele and blue lines for the maternal allele. The allelic DNA methylation levels (mCG/CG) are shown with red lines for the paternal allele and blue lines for the maternal allele. The pDMRs identified are highlighted by the dashed boxes.

To investigate the conservation of allelic DNA methylation patterns in different reciprocal hybrids, we next performed MethylC-seq for the endosperm of MC/CM crosses. The average methylation levels of CG, CHG, and CHH were 69.2%, 45.3%, and 1.3%, respectively, in the CM endosperm and 71.6%, 43.5%, and 1.1%, respectively, in the endosperm of MC, respectively (Supplementary Fig. S3). We scanned the genomes in the MC/CM crosses for parent-of-origin dependent differentially methylated regions (pDMRs) using a sliding window strategy, and this resulted in the identification of 1367 pDMRs in the CG context (CG_pDMRs) and 334 pDMRs in the CHG context (CHG_pDMRs) in the MC/CM endosperm (Supplementary Table S7). Most CHG_pDMRs overlapped with CG_pDMRs in MC/CM (Supplementary Fig. S4), which matched a previous result in the endosperm of BM/MB (Zhang *et al.*, 2014). Compared to all the analysed regions in the CG context, the pDMRs tended to be located in genetic regions (Supplementary Fig. S5). We next compared the allelic methylation patterns at the pDMR regions identified in the endosperm of MC/CM and BM/MB by acquiring published whole-genome bisulfite sequencing data from endosperm samples of BM and MB (Zhang *et al.*, 2014). This indicated that 84.3% of CM/MC pDMRs in the CG context showed significant maternal hypomethylation and paternal hypermethylation in BM/MB endosperm (*P*<0.01). Meanwhile, 88.67% of BM/MB pDMRs in the CG context also showed maternal demethylation in CM/MC endosperm (*P*<0.01; Fig. 3A–C). We termed these pDMRs conserved CG_pDMRs in BM/MB and MC/CM endosperm. However, some (~15%) BM/MB or MC/CM non-conserved CG_pDMRs were identified. Similar results were obtained in the CHG context (Supplementary Fig. S6).

### The association of imprinted genes and DNA methylation in BM/MB and MC/CM endosperm

MEGs and PEGs had different patterns of allele-specific DNA methylation in MC/CM endosperm. In the CG context, we found that compared with all genes, the methylation levels of the maternal alleles of MEGs were slightly lower than those of the paternal alleles in the 5´ portion of the gene body regions (Fig. 3D, F). However, the DNA methylation levels of the maternal alleles of PEGs were clearly lower than those of their paternal alleles along their upstream and gene-body regions (Fig. 3E, F). For the CHG context, slightly lower maternal DNA methylation was also found at the 5´ portions of the gene bodies of MEGs (Fig. 3G, I) whilst for PEGs, lower maternal DNA methylation was observed only in the upstream regions (Fig. 3H, I).

The availability of identified imprinted genes together with whole-genome DNA methylome data in the MC/CM and BM/MB crosses allowed us to investigate the relationship between epigenetic variation and allelic imprinting in maize. First, we analysed the association of conserved CG_pDMRs with imprinted genes, and found that BM/MB conserved CG_pDMRs were located in 77 imprinted genes (11 MEGs and 66 PEGs) identified in the BM/MB endosperm. Among the 71 genes that were analysed allelically, 64 (90.1%) were imprinted in MC/CM endosperm (Supplementary Fig. S7). Similarly, the MC/CM conserved CG_pDMRs were located at 17 imprinted genes identified in MC/CM endosperm. Among the 16 genes analysed allelically, 15 (93.7%) were imprinted in BM/MB endosperm. Hence, conserved pDMRs tended to be associated with intraspecifically conserved imprinted genes in maize (Supplementary Table S7).

The association between BM/MB- or MC/CM-specific CG_pDMRs and BM/MB- or MC/CM-specific imprinted genes was then investigated. The BM/MB-specific CG_pDMRs were located at four PEGs (*Zm00001d032655*, *Zm00001d025021*, *Zm00001d003864*, *Zm00001d041290*) identified in the BM/MB endosperm. Among these four genes, *Zm00001d003864* was a non-imprinted gene, and the other three were not allelically analysed in the MC/CM endosperm. The MC/CM-specific CG_pDMRs were located at one PEG (*Zm00001d038329*) and one MEG (*Zm00001d051877*) identified in the MC/CM endosperm. *Zm00001d038329* was a non-imprinted gene, and *Zm00001d051877* was not allelically analysed in the BM/MB endosperm. The integrated profiles of allele-specific DNA methylation and gene expression in BM/MB and MC/CM endosperm are shown in Fig. 3J, K at *Zm00001d003864* (PEG in BM/MB but non-imprinted in MC/CM) and *Zm00001d038329* (PEG in MC/CM but non-imprinted in BM/MB). These results seemed to indicate that the variation in DNA methylation might be associated with the variation in imprinting of these genes in maize.

### Phenotype analysis of intraspecific conserved imprinted genes in knockout lines

**Fig. 4.**
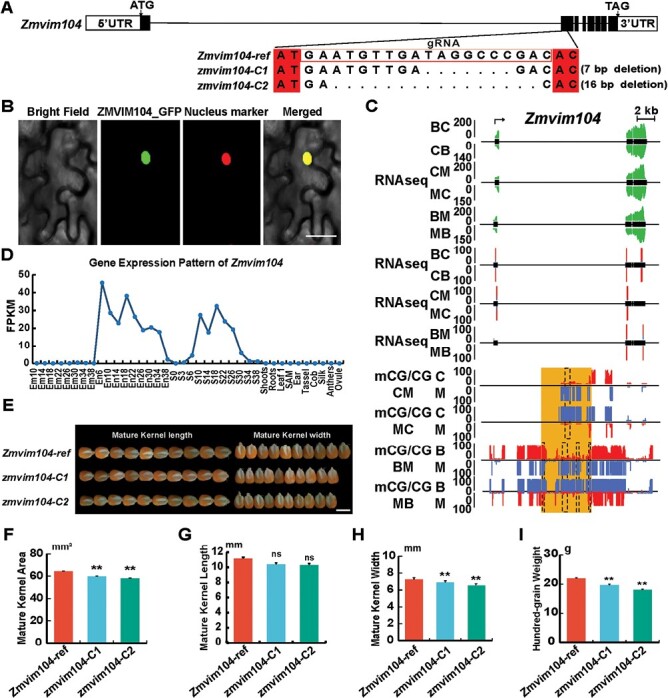
Phenotype analysis of *Zmvim104*. (A) Diagram of the two CRISPR/Cas9 lines of *Zmvim104* compared with the reference in the KN5585 maize receptor line. (B) Subcellular localization of the full-length Zmvim104-GFP fusion protein in tobacco leaf epidermal cells. The nuclear marker was P2300-35S-H2B-mCherry. The scale bar is 20 μm. (C) Integrated profiles of allele-specific gene expression and DNA methylation levels of *Zmvim104* in the endosperm of the different crosses (Table 1). The expression levels according to RNA-seq are shown in green, and the percentages of allelic reads for specific SNP sites are shown with red lines for the paternal allele and blue lines for the maternal allele. The allelic DNA methylation levels (mCG/CG) are shown with red lines for the paternal allele and blue lines for the maternal allele. The dashed boxes highlight the differentially methylated regions (DMRs) that were dependent on the parent of origin (pDMRs) for the CG sites. (D) Expression pattern of *Zmvim104* in different tissues of KN5585 during plant development. The age of each tissue is given in days. Em, embryo; En, endosperm; S, seed; SAM, stem apical meristem. (E) Kernel phenotypes of the KN5585 maize receptor line and the two CRISPR/Cas lines. The scale bar is 1 cm. (F–I) Quantification of the kernel phenotypes: (F) kernel area, (G) kernel length, (H) kernel width, and (I) hundred-grain weight. Data are means (±SD) of 30 biological replicates. Significant differences compared with the reference line were determined using Student’s two-tailed *t*-tests: **P<0.01; ns, not significant.

An intraspecifically conserved PEG, *Zm00001d019342*, which is a variant of methylation 104 (denoted as *Zmvim104*), was selected to further study the gene function in kernel development. Using CRISPR/Cas9 technology, two *Zmvim104* frame-shift mutant lines were created for phenotype analysis (*zmvim104-C1* with a 7 bp deletion, and *zmvim104-C2* with 16 bp deletion; Fig. 4A). We first investigated the subcellular localization of the Zmvim104 protein in tobacco leaf epidermal cells and found that the full-length Zmvim104-GFP fusion protein was localized to the nucleus (Fig. 4B). Conserved pDMRs were found at the promoter region of *Zmvim104* in MC/CM and BM/MB endosperm (Fig. 4C). Expression analysis showed that *Zmvim104* was highly expressed in kernels (Fig. 4D), and hence we focused on kernel phenotypes between the knockout lines and the control line (Fig. 4E). Surprisingly, the mature kernel area of both the mutant lines was significantly smaller than that of the control line (Fig. 4F). There were no notable differences in the kernel length (Fig. 4G), but the widths of the two mutant lines were significantly smaller than the control line (Fig. 4H). In addition, the hundred-grain weights of two mutant lines were smaller than the control line (Fig. 4I), which indicated that *Zmvim104* might play an important role in kernel development.

**Fig. 5.**
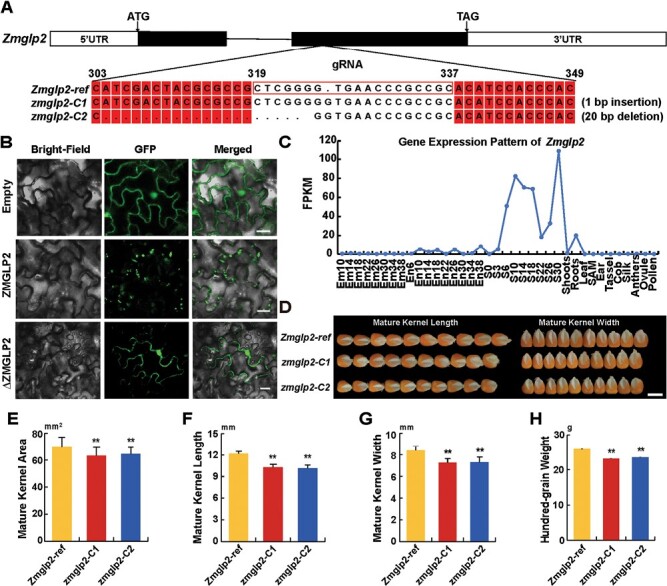
Phenotype analysis of *Zmglp2*. (A) Diagram of the two CRISPR/Cas9 lines of *Zmglp2* compared with the reference in the KN5585 maize receptor line (B) Subcellular localization of the full-length and truncated Zmglp2-GFP fusion proteins in tobacco leaf epidermal cells. The truncated protein (∆Zmglp2) protein had amino acids 2–24 removed. The empty vector with GFP alone was used as a control. Scale bars are 20 μm. (C) Expression pattern of *Zmmglp2* in different tissues of KN5585 during plant development. The age of each tissue is given in days. Em, embryo; En, endosperm; S, seed; SAM, stem apical meristem. (D) Kernel phenotypes of the KN5585 maize receptor line and the two CRISPR/Cas lines. The scale bar is 1 cm. (E–H) Quantification of the kernel phenotypes: (E) kernel area, (F) kernel length, (G) kernel width, and (H) hundred-grain weight. Data are means (±SD) of 30 biological replicates. Significant differences compared with the reference line were determined using two-tailed Student’s *t*-tests: **P<0.01.

As previously reported, MEGs are likely to be involved in nutrient transport and hormone signaling (Xin *et al.*, 2013; Zhang *et al.*, 2014). To study gene function in kernel development, we selected a MEG that was intraspecifically conserved imprinted in maize, namely *Zm00001d004401*, which encodes a germin-like protein2 (hence denoted as *Zmglp2*) ,. Using CRISPR/Cas9 technology, we created two *zmglp2* mutant lines (*zmglp2*-C1 with a 1 bp insertion, and *zmglp2*-C2 with a 20 bp deletion; Fig. 5A). In tobacco leaf epidermal cells, the full-length Zmglp2 protein was localized in the extracellular space or cell membrane (Fig. 5B), indicating that it might act as a secreted protein. The truncated ∆Zmglp2-GFP fusion protein (2–24 aa removed) was found to be localized in the nucleus and the cell membrane, indicating that the signal peptide sequence might be located at 2–24 aa. We then examined the *Zmglp2* expression pattern and found that it was similar to that of *Zmvim104* (Fig. 5C). Interestingly, the two *zmglp2* mutant lines showed similar phenotypes to the *zmvim104* mutants (Fig. 5D–H), although the kernel length was as well as the width was reduced in the *zmglp2* mutants.

### Interspecific conservation of imprinted genes

To study the interspecific conservation of the imprinted genes that we identified in the three reciprocal hybrids in maize, we compared the imprinting status of the conserved and non-conserved genes in sorghum, rice, and Arabidopsis (Supplementary Table S8). Among the 275intraspecies-conserved imprinted genes (Supplementary Table S5), 16 MEGs and 47 PEGs were also imprinted in rice (25 genes), sorghum (34 genes), and Arabidopsis (seven genes). Among the 147intraspecifically non-conserved imprinted genes, three MEGs and nine PEGs were imprinted in rice (nine genes), sorghum (two genes) and Arabidopsis (one gene). In total, 22.9% (63 out of 275) of intraspecies-conserved imprinted genes were found to be interspecifically conserved, which was significantly higher than the rate of intraspecific non-conserved imprinted genes, which was 8.2% (12 out of 147) (*P*=0.0005). In addition, we also found some of the genes showing an opposite imprinting status interspecifically not only in the conserved part but also in the non-conserved part.

## Discussion

### Variation in DNA methylation plays an important role in intraspecific variation in imprinting

**Fig. 6.**
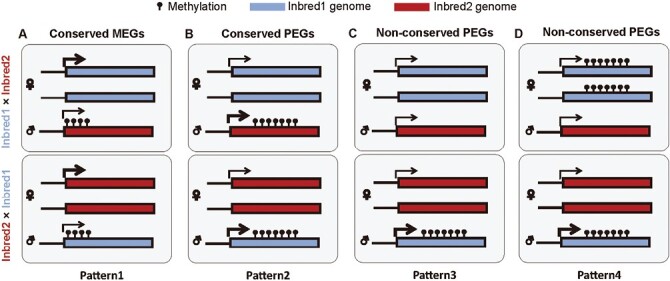
Possible allelic methylation patterns of conserved and non-conserved imprinted genes in embryos of intraspecies crosses. (A) Model for conserved maternally expressed genes (MEGs). In inbred1 × inbred2 and inbred2 × inbred1, maternal DNA demethylation around the upstream and gene body regions leads to maternal-specific expression. (B) Model for conserved paternally expressed genes (PEGs). In inbred1 × inbred2 and inbred2 × inbred1, maternal DNA demethylation within the gene body potentially leads to maternal-specific H3K27me3 modification and repression of maternal expression. (C, D) Two different patterns are observed for non-conserved PEGs. (C) In inbred1 × inbred2, there are no differences in methylation in the maternal and paternal gene bodies, and hence both alleles can be expressed. But in inbred2 × inbred1, maternal DNA demethylation within the gene body potentially leads to maternal-specific H3K27me3 modification and repression of maternal expression. (D) In inbred1 × inbred2, there is hypermethylation level in the maternal gene body and hypomethylation in the paternal gene body, and the maternal and paternal alleles can be expressed. But in inbred2 × inbred1, maternal DNA demethylation within the gene body potentially leads to maternal-specific H3K27me3 modification and repression of maternal expression. The thickness of the arrows denotes the relative expression level. Methylation refers to methylated cytosine.

In the maize reciprocal crosses that we studied (Table 1), nearly 15% of the parent-of-origin dependent differentially methylated regions (pDMRs) were intraspecifically non-conserved (Fig. 3C). Unexpectedly, the percentage was well matched with that of imprinted genes, showing evidence of allele variation for intraspecifical imprinting in maize, which indicates that DNA methylation variants play an important role in imprinting variants. However, some conserved pDMRs were still located at non-conserved imprinted genes. Therefore, other epigenetic marks are probably involved in the epigenetic regulation of allelic imprinting variants, such as active or repressive histone modifications. In this study, we found that the allelic imprinting expression of two imprinted genes was associated with variants of DNA methylation. *Zm00001d038329* is annotated as transmembrane protein 18, and it was a paternally expressed gene (PEG) in endosperm from the MC, CM, and MB crosses but was is biallelically expressed in endosperm from BM crosses (Fig. 3K). Maternal demethylation was observed in MC/CM-specific pDMRs in the MB cross, while hypermethylation was observed in maternal and paternal alleles from the BM cross. The removal of methylation by DEMETER (DME) is not random and occurs in small, euchromatic, nucleosome-poor, AT-rich transposons (Ibarra *et al.*, 2012). Therefore, the sequence variants might contribute to DME not being able to bind to *Zm00001d038329* in the B73 line. *Zm00001d003864* is annotated as a homolog of *SNF4* in Arabidopsis, which is part of the regulatory complex that promotes kinase activity. The expression of *Zm00001d003864* was paternally biased in endosperm from BM, MB, MC, and BC crosses, but was biallelically expressed in endosperm from CM and CB crosses (Fig. 3J). In the region of BM/MB-specific pDMRs at *Zm00001d003864*, maternal demethylation was observed in MC crosses, while hypomethylation was observed at both the maternal and paternal alleles from CM crosses. Based on the above results, we summarize the possible allelic methylation pattern of intraspecifically conserved and non-conserved imprinted genes in Fig. 6.

### Conserved imprinted genes might play important roles in the plant development process

In our study, intraspecifically conserved imprinted genes were enriched in some specific pathways (Fig. 2). In the cellular component pathway, conserved imprinted genes were highly enriched in the nucleus category, which is consistent with previous results in Arabidopsis showing that most imprinted genes are located in the nucleus (Raissig *et al.*, 2013), and indicates that these genes might play an important role in the kernel development process. Hence, conserved imprinted genes might be considered as a research priority to determine their functional roles in seed development by reverse-genetic analysis. Indeed, in this study we found that the mutation of two imprinted genes (one MEG and one PEG) influenced the development of the kernel (Figs 4, 5). There are currently three main theories for the evolution of genomic imprinting, namely sexual antagonism, maternal-offspring co-adaptation, and parental conflict (also known as the kinship theory; Patten *et al.*, 2014). In maize, the imprinted *dosage-effect defective1* (*ded1*) locus provides critical support for the parental conflict theory. The paternally inherited *Ded1* allele is sufficient to promote embryo development and normal seed weight while the maternal allele is sufficient for seed development but with a reduced weight, which indicates that the paternal allele increases the uptake of nutritional resources and the accumulation of seed reserves (Dai *et al.*, 2022). On the other hand, *Maternally expressed gene1* (*Meg1*) provides an important example in favour of the maternal-offspring co-adaptation theory. Maternally expressed *Meg1* in maize positively regulates the development and function of transfer tissue, thereby promoting the nutrient-uptake capacity of the seed and ultimately increasing yield (Costa *et al.*, 2012). Hence, further research on the qualitative seed phenotypes of the two imprinted genes that we identified should be carried out in the future. In our study, disruption of *Zmvim104* (a PEG) by CRISPR/Cas9 resulted in a phenotype with a smaller kernel. Under the parental conflict theory, inbred lines producing small seeds could be due to being more maternalized, and thus *Zmvim104* might provide an example in support of the parental conflict theory. In addition, disruption of *Zmglp2* (a MEG) by CRISPR/Cas9 also resulted in a smaller kernel phenotype, and thus it might provide an example in support of the maternal-offspring co-adaptation theory, like *Meg1*.

### Tissue-specific imprinted genes in the embryo

We assessed potential contamination of 18 embryo transcriptomes according to the statistical tool presented by Schon and Nodine (2017). We found that all 18 embryo transcriptomes were highly enriched for the embryonic tissue isolated by the LCM technique in a previous study (Zhan *et al.*, 2015), and they were not significantly contaminated by RNA from maternal or endosperm tissues (Supplementary Fig. S8). In addition, one embryo-specific MEG in our study (*Zm00001d030305*) has been tested using RNA hybridization in a previous study (Meng *et al.*, 2018), and this helped confirm that our embryo tissues were clean of other seed tissues, and hence that our RNA-seq libraries were reliable in terms of identifying the parental bias of genes.

The embryo and endosperm are the products of double-fertilization, and the paternal genomes of both are derived from the same spermatids. We focused on the tissue-specific genes detected in our results. Among 34 imprinted genes identified in the embryo, 22 (64.7%) were embryo-specific imprinted genes (Supplementary Table S5). We found that 12 imprinted genes in the embryo were also imprinted in the endosperm. Interestingly, three MEGs (*Zm00001d020055*, *Zm00001d020769*, and *Zm00001d022443*) in the embryo were PEGs in the endosperm. According to previous studies, one of the imprinting mechanisms, DNA methylation, is the opposite in the embryo and endosperm (methylation and demethylation, respectively), and the methylation level of the endosperm is lower than that of the embryo (Gehring *et al.*, 2009; Hsieh *et al.*, 2009; Zhang *et al.*, 2014). In addition, the different types of imprinting in the embryo and endosperm might also be affected by histone modifications, long non-coding RNA, or other mechanisms, and this requires further research.

### Conclusions

Our study showed that most of the imprinted genes in our crosses exhibited intraspecific conservation, and nearly half of these genes also exhibited interspecific conservation and were enriched in some regulation pathways. Disruption of two conserved imprinted genes using CRISPR/Cas9 editing resulted in a phenotype with smaller kernels and lower hundred-grain weight. Whole-genome DNA methylation sequencing revealed that nearly 15% of DNA methylation existed as allelic variants, and these variants might play an important role in affecting imprinting status. In future work, we aim to conduct more detailed functional analysis of the interspecific and intraspecific conserved genes detected in this study.

## Supplementary data

The following supplementary data are available at *JXB* online.

Fig. S1. Cluster dendrogram showing global transcriptome relationships in the embryo and endosperm.

Fig. S2. Verification of imprinted genes by PCR sequencing.

Fig. S3. The bulk DNA methylation levels in the endosperm of the CM and MC crosses.

Fig. S4. The overlap between CG_pDMR and CHG_pDMR in MC/CM endosperm.

Fig. S5. The genomic distributions of CG_pDMRs.

Fig. S6. Comparison of allele methylation in the CHG_pDMR regions identified in MC/CM and BM/MB endosperm.

Fig. S7. The overlap between conserved pDMRs and imprinted genes in MC/CM and BM/MB endosperm.

Fig. S8. Heat-map illustrating results from tissue enrichment tests on embryo and endosperm transcriptomes from three reciprocal crosses.

Table S1. List of primers used in the study.

Table S2. List of KASP markers used for haploid detection.

Table S3. Size and quality of data obtained in the study.

Table S4. Correlations of the results among the three replicates in the embryo and endosperm in each cross.

Table S5. List of the imprinted genes detected in the maize embryo and endosperm.

Table S6. Clusters of the imprinted genes detected in our results in the maize genome.

Table S7. pDMRs in the CG and CHG contexts in CM/MC endosperm.

Table S8. Interspecific conservation of imprinted genes.

erad440_suppl_Supplementary_Tables_S1-S8

erad440_suppl_Supplementary_Figures_S1-S9

## Data Availability

Sequence data from this study can be found in the Sequence Read Archive at NCBI (https://www.ncbi.nlm.nih.gov/sra) under accession number PRJNA765150. All other data supporting the findings of this study are available within the paper and within its supplementary data published online.

## Abbreviations

DAP: days after pollination
MEG: maternally expressed gene
PEG: paternally expressed gene
WT: wild type

## References

[CIT0001] Bernardi J , Lanubile A, Li QB, Kumar D, Kladnik A, Cook SD, Ross JJ, Marocco A, Chourey PS. 2012. Impaired auxin biosynthesis in the *defective endosperm18* mutant is due to mutational loss of expression in the *ZmYuc1* gene encoding endosperm-specific YUCCA1 protein in maize. Plant Physiology 160, 1318–1328. 22961134 10.1104/pp.112.204743 PMC3490580

[CIT0002] Chaudhury AM , Koltunow A, Payne T, Luo M, Tucker MR, Dennis ES, Peacock WJ. 2001. Control of early seed development. Annual Review of Cell and Developmental Biology 17, 677–699. 10.1146/annurev.cellbio.17.1.677 11687501

[CIT0003] Costa LM , Yuan J, Rouster J, Paul W, Dickinson H, Gutierrez-Marcos JF. 2012. Maternal control of nutrient allocation in plant seeds by genomic imprinting. Current Biology 22, 160–165. 22245001 10.1016/j.cub.2011.11.059

[CIT0004] Cox MP , Peterson DA, Biggs PJ. 2010. SolexaQA: At-a-glance quality assessment of Illumina second-generation sequencing data. BMC Bioinformatics 11, 485. 20875133 10.1186/1471-2105-11-485 PMC2956736

[CIT0005] Dai D , Mudunkothge JS, Galli M, et al. 2022. Paternal imprinting of *dosage-effect defective1* contributes to seed weight xenia in maize. Nature Communications 13, 5366. 10.1038/s41467-022-33055-9 PMC9470594 36100609

[CIT0006] Dumas C , Mogensen HL, Ishida Y, Hiei Y, Komari T. 1993. Gametes and fertilization: maize as a model system for experimental embryogenesis in flowering plants. The Plant Cell 5, 1337–1348. 12271033 10.1105/tpc.5.10.1337 PMC160366

[CIT0007] Gehring M , Bubb KL, Henikoff S. 2009. Extensive demethylation of repetitive elements during seed development underlies gene imprinting. Science 324, 1447–1451. 19520961 10.1126/science.1171609 PMC2886585

[CIT0008] Guo H , Cao P, Wang C, et al. 2023. Population analysis reveals the roles of DNA methylation in tomato domestication and metabolic diversity. Science China Life Sciences 66, 1888–1902. 36971992 10.1007/s11427-022-2299-5

[CIT0009] Hatorangan MR , Laenen B, Steige KA, Slotte T, Köhler C. 2016. Rapid evolution of genomic imprinting in two species of the Brassicaceae. The Plant Cell 28, 1815–1827. 27465027 10.1105/tpc.16.00304 PMC5006707

[CIT0010] Hsieh TF , Ibarra CA, Silva P, Zemach A, Eshed-Williams L, Fischer RL, Zilberman D. 2009. Genome-wide demethylation of Arabidopsis endosperm. Science 324, 1451–1454. 19520962 10.1126/science.1172417 PMC4044190

[CIT0011] Ibarra CA , Feng X, Schoft VK, et al. 2012. Active DNA demethylation in plant companion cells reinforces transposon methylation in gametes. Science 337, 1360–1364. 22984074 10.1126/science.1224839 PMC4034762

[CIT0012] Ishida Y , Hiei Y, Komari T. 2007. *Agrobacterium*-mediated transformation of maize. Nature Protocols 2, 1614–1621. 17585302 10.1038/nprot.2007.241

[CIT0013] Iwasaki M , Hyvärinen L, Piskurewicz U, Lopez-Molina L. 2019. Non-canonical RNA-directed DNA methylation participates in maternal and environmental control of seed dormancy. eLife 8, e37434. 30910007 10.7554/eLife.37434 PMC6435323

[CIT0014] Kawakatsu T , Huang SC, Jupe F, et al. 2016. Epigenomic diversity in a global collection of *Arabidopsis thaliana* accessions. Cell 166, 492–505. 27419873 10.1016/j.cell.2016.06.044 PMC5172462

[CIT0015] Kermicle JL. 1970. Dependence of the *R*-mottled aleurone phenotype in maize on mode of sexual transmission. Genetics 66, 69–85. 17248508 10.1093/genetics/66.1.69 PMC1212486

[CIT0016] Kim D , Paggi JM, Park C, Bennett C, Salzberg SL. 2019. Graph-based genome alignment and genotyping with HISAT2 and HISAT-genotype. Nature Biotechnology 37, 907–915. 10.1038/s41587-019-0201-4 PMC7605509 31375807

[CIT0017] Krueger F , Andrews SR. 2011. Bismark: a flexible aligner and methylation caller for bisulfite-seq applications. Bioinformatics 27, 1571–1572. 21493656 10.1093/bioinformatics/btr167 PMC3102221

[CIT0018] Lafon-Placette C , Hatorangan MR, Steige KA, Cornille A, Lascoux M, Slotte T, Köhler C. 2018. Paternally expressed imprinted genes associate with hybridization barriers in *Capsella*. Nature Plants 4, 352–357. 29808019 10.1038/s41477-018-0161-6

[CIT0019] Li H , Durbin R. 2009. Fast and accurate short read alignment with Burrows–Wheeler transform. Bioinformatics 25, 1754–1760. 19451168 10.1093/bioinformatics/btp324 PMC2705234

[CIT0020] Li H , Handsaker B, Wysoker A, Fennell T, Ruan J, Homer N, Marth G, Abecasis G, Durbin R; 1000 Genome Project Data Processing Subgroup. 2009. The sequence alignment/map format and SAMtools. Bioinformatics 25, 2078–2079. 19505943 10.1093/bioinformatics/btp352 PMC2723002

[CIT0021] Lu X , Chen D, Shu D, Zhang Z, Wang W, Klukas C, Chen LL, Fan Y, Chen M, Zhang C. 2013. The differential transcription network between embryo and endosperm in the early developing maize seed. Plant Physiology 162, 440–455. 23478895 10.1104/pp.113.214874 PMC3641222

[CIT0022] Martinez G , Wolff P, Wang Z, Moreno-Romero J, Santos-González J, Conze LL, DeFraia C, Slotkin RK, Köhler C. 2018. Paternal easiRNAs regulate parental genome dosage in Arabidopsis. Nature Genetics 50, 193–198. 29335548 10.1038/s41588-017-0033-4

[CIT0023] Meng D , Zhao J, Zhao C, Luo H, Xie M, Liu R, Lai J, Zhang X, Jin W. 2018. Sequential gene activation and gene imprinting during early embryo development in maize. The Plant Journal 93, 445–459. 29172230 10.1111/tpj.13786

[CIT0024] Monk D , Mackay DJG, Eggermann T, Maher ER, Riccio A. 2019. Genomic imprinting disorders: lessons on how genome, epigenome and environment interact. Nature Reviews Genetics 20, 235–248. 10.1038/s41576-018-0092-0 30647469

[CIT0025] Patten MM , Ross L, Curley JP, Queller DC, Bonduriansky R, Wolf JB. 2014. The evolution of genomic imprinting: theories, predictions and empirical tests. Heredity 113, 119–128. 24755983 10.1038/hdy.2014.29 PMC4105453

[CIT0026] Pignatta D , Erdmann RM, Scheer E, Picard CL, Bell GW, Gehring M. 2014. Natural epigenetic polymorphisms lead to intraspecific variation in Arabidopsis gene imprinting. eLife 3, e03198. 24994762 10.7554/eLife.03198 PMC4115658

[CIT0027] Pignatta D , Novitzky K, Satyaki PRV, Gehring M. 2018. A variably imprinted epiallele impacts seed development. PLoS Genetics 14, e1007469. 30395602 10.1371/journal.pgen.1007469 PMC6237401

[CIT0028] Piskurewicz U , Iwasaki M, Susaki D, Megies C, Kinoshita T, Lopez-Molina L. 2016. Dormancy-specific imprinting underlies maternal inheritance of seed dormancy in *Arabidopsis thaliana*. eLife 5, e19573. 28005006 10.7554/eLife.19573 PMC5243116

[CIT0029] Raissig MT , Bemer M, Baroux C, Grossniklaus U. 2013. Genomic imprinting in the Arabidopsis embryo is partly regulated by PRC2. PLoS Genetics 9, e1003862. 24339783 10.1371/journal.pgen.1003862 PMC3854695

[CIT0030] Rodrigues JA , Ruan R, Nishimura T, Sharma MK, Sharma R, Ronald PC, Fischer RL, Zilberman D. 2013. Imprinted expression of genes and small RNA is associated with localized hypomethylation of the maternal genome in rice endosperm. Proceedings of The National Academy of Sciences, USA 110, 7934–7939. 10.1073/pnas.1306164110 PMC3651473 23613580

[CIT0031] Schmitz RJ , Schultz MD, Urich MA, et al. 2013. Patterns of population epigenomic diversity. Nature 495, 193–198. 23467092 10.1038/nature11968 PMC3798000

[CIT0032] Schon MA , Nodine MD. 2017. Widespread contamination of Arabidopsis embryo and endosperm transcriptome data sets. The Plant Cell 29, 608–617. 28314828 10.1105/tpc.16.00845 PMC5435428

[CIT0033] Tian T , Liu Y, Yan H, You Q, Yi X, Du Z, Xu W, Su Z. 2017. agriGO v20: a GO analysis toolkit for the agricultural community, 2017 update. Nucleic Acids Research 45, W122–W129. 28472432 10.1093/nar/gkx382 PMC5793732

[CIT0034] Trapnell C , Roberts A, Goff L, Pertea G, Kim D, Kelley DR, Pimentel H, Salzberg SL, Rinn JL, Pachter L. 2012. Differential gene and transcript expression analysis of RNA-seq experiments with TopHat and Cufflinks. Nature Protocols 7, 562–578. 22383036 10.1038/nprot.2012.016 PMC3334321

[CIT0035] Waters AJ , Bilinski P, Eichten SR, Vaughn MW, Ross-Ibarra J, Gehring M, Springer NM. 2013. Comprehensive analysis of imprinted genes in maize reveals allelic variation for imprinting and limited conservation with other species. Proceedings of The National Academy of Sciences, USA 110, 19639–19644. 10.1073/pnas.1309182110 PMC3845156 24218619

[CIT0036] Xin M , Yang R, Li G, et al. 2013. Dynamic expression of imprinted genes associates with maternally controlled nutrient allocation during maize endosperm development. The Plant Cell 25, 3212–3227. 24058158 10.1105/tpc.113.115592 PMC3809528

[CIT0037] Xing HL , Dong L, Wang ZP, Zhang HY, Han CY, Liu B, Wang XC, Chen QJ. 2014. A CRISPR/Cas9 toolkit for multiplex genome editing in plants. BMC Plant Biology 14, 327. 25432517 10.1186/s12870-014-0327-y PMC4262988

[CIT0038] Xu G , Lyu J, Li Q, Liu H, Wang D, Zhang M, Springer NM, Ross-Ibarra J, Yang J. 2020. Evolutionary and functional genomics of DNA methylation in maize domestication and improvement. Nature Communications 11, 5539. 10.1038/s41467-020-19333-4 PMC7606521 33139747

[CIT0039] Yang L , Xing F, He Q, Tahir Ul Qamar M, Chen LL, Xing Y. 2020. Conserved imprinted genes between intra-subspecies and inter-subspecies are involved in energy metabolism and seed development in rice. International Journal of Molecular Sciences 21, 9618. 33348666 10.3390/ijms21249618 PMC7765902

[CIT0040] Zhan J , Thakare D, Ma C, et al. 2015. RNA sequencing of laser-capture microdissected compartments of the maize kernel identifies regulatory modules associated with endosperm cell differentiation. The Plant Cell 27, 513–531. 25783031 10.1105/tpc.114.135657 PMC4558669

[CIT0041] Zhang M , Li N, He W, Zhang H, Yang W, Liu B. 2016. Genome-wide screen of genes imprinted in sorghum endosperm, and the roles of allelic differential cytosine methylation. The Plant Journal 85, 424–436. 26718755 10.1111/tpj.13116

[CIT0042] Zhang M , Xie S, Dong X, et al. 2014. Genome-wide high resolution parental-specific DNA and histone methylation maps uncover patterns of imprinting regulation in maize. Genome Research 24, 167–176. 24131563 10.1101/gr.155879.113 PMC3875858

[CIT0043] Zhu H , Xie W, Xu D, Miki D, Tang K, Huang CF, Zhu JK. 2018. DNA demethylase ROS1 negatively regulates the imprinting of *DOGL4* and seed dormancy in *Arabidopsis thaliana*. Proceedings of The National Academy of Sciences, USA 115, E9962–E9970. 10.1073/pnas.1812847115 PMC6196528 30266793

[CIT0044] Zuo Y , Feng F, Qi W, Song R. 2019. *Dek42* encodes an RNA-binding protein that affects alternative pre-mRNA splicing and maize kernel development. Journal of Integrative Plant Biology 61, 728–748. 30839161 10.1111/jipb.12798

