# Supplementary material for: The conservation of allelic DNA methylation and its relationship with imprinting in maize
Source: J Exp Bot. 2023 Nov 3;75(5):1376–89. doi: 10.1093/jxb/erad440 (PMC10901201; doi:10.1093/jxb/erad440)
Supplement: erad440_suppl_Supplementary_Figures_S1-S9 [file erad440_suppl_supplementary_figures_s1-s9.pdf]

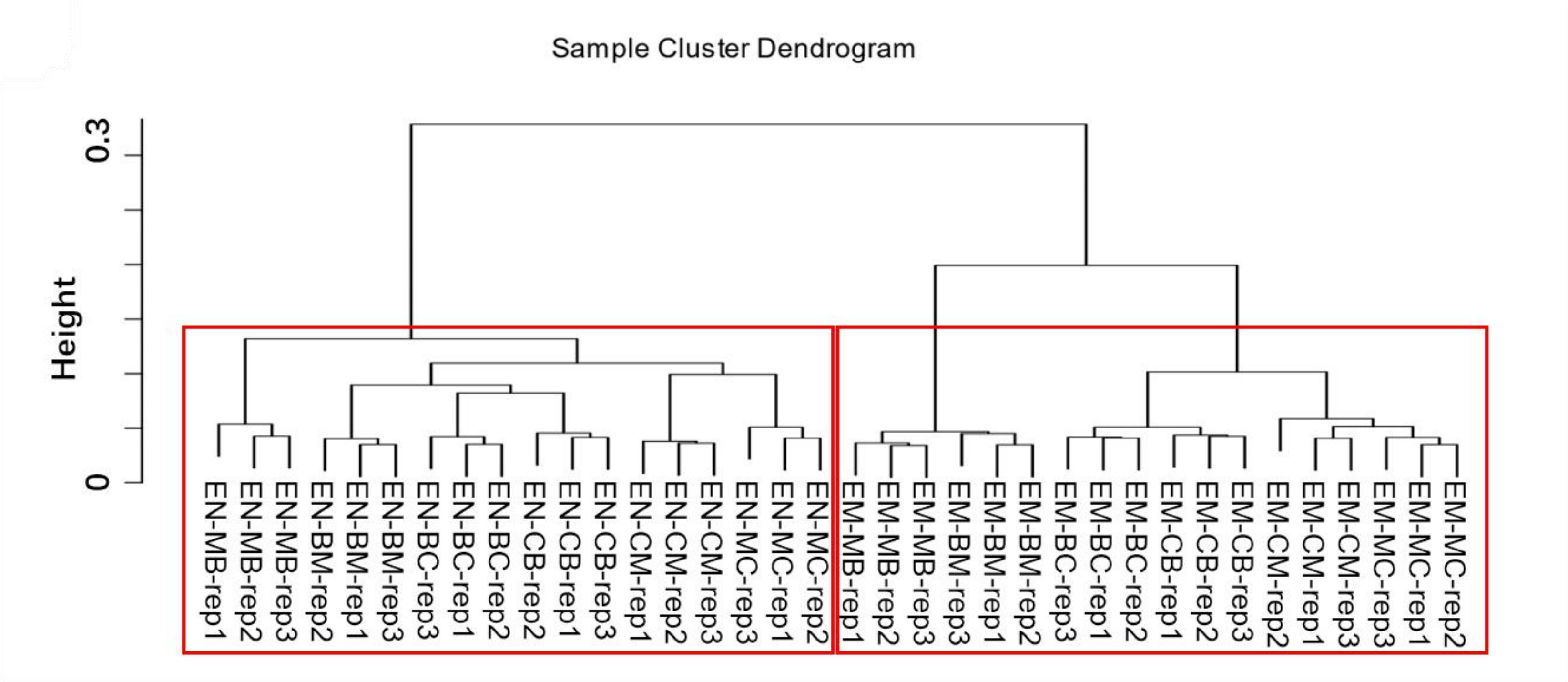

Fig. S1. Cluster dendrogram showing global transcriptome relationships among in the embryo and endosperm.

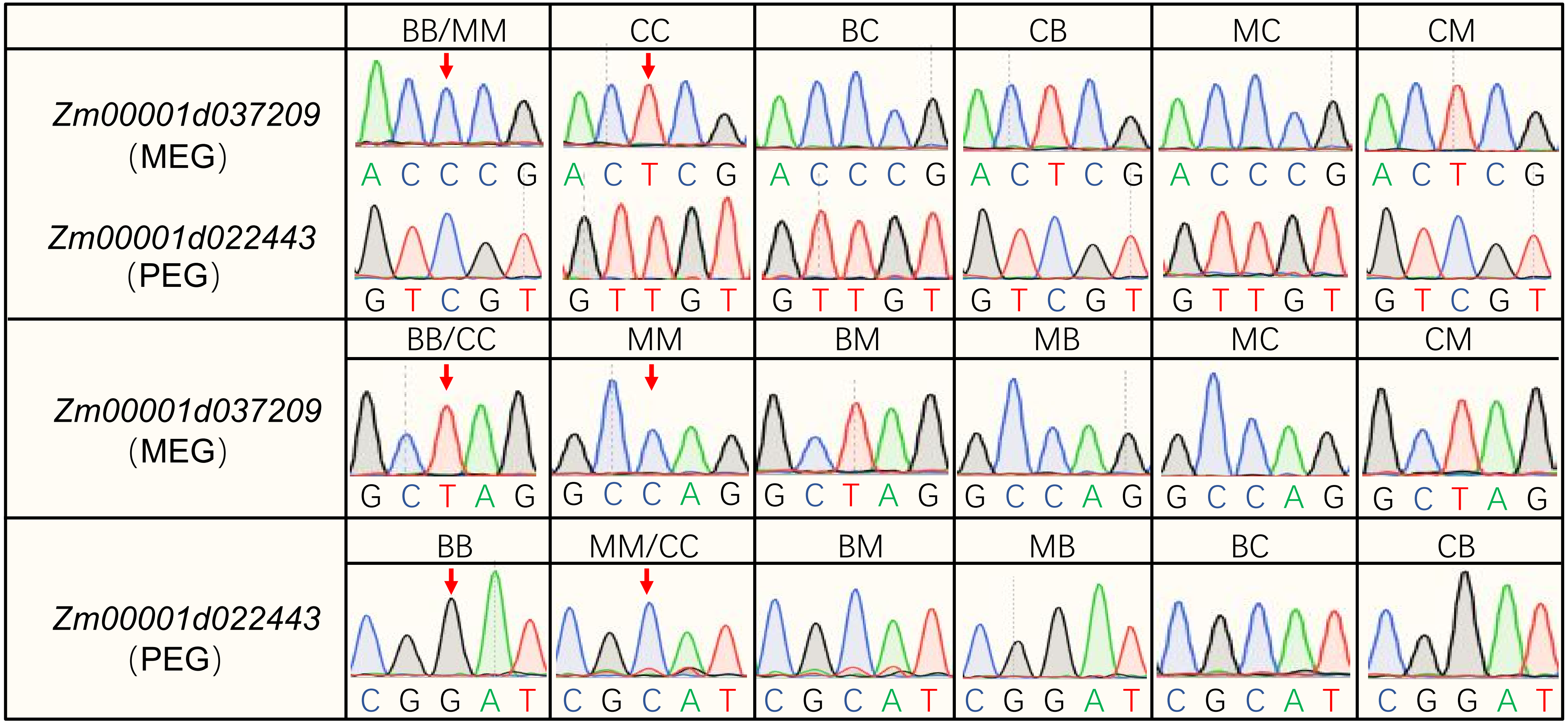

Fig. S2. Verification of imprinted genes by PCR sequencing.

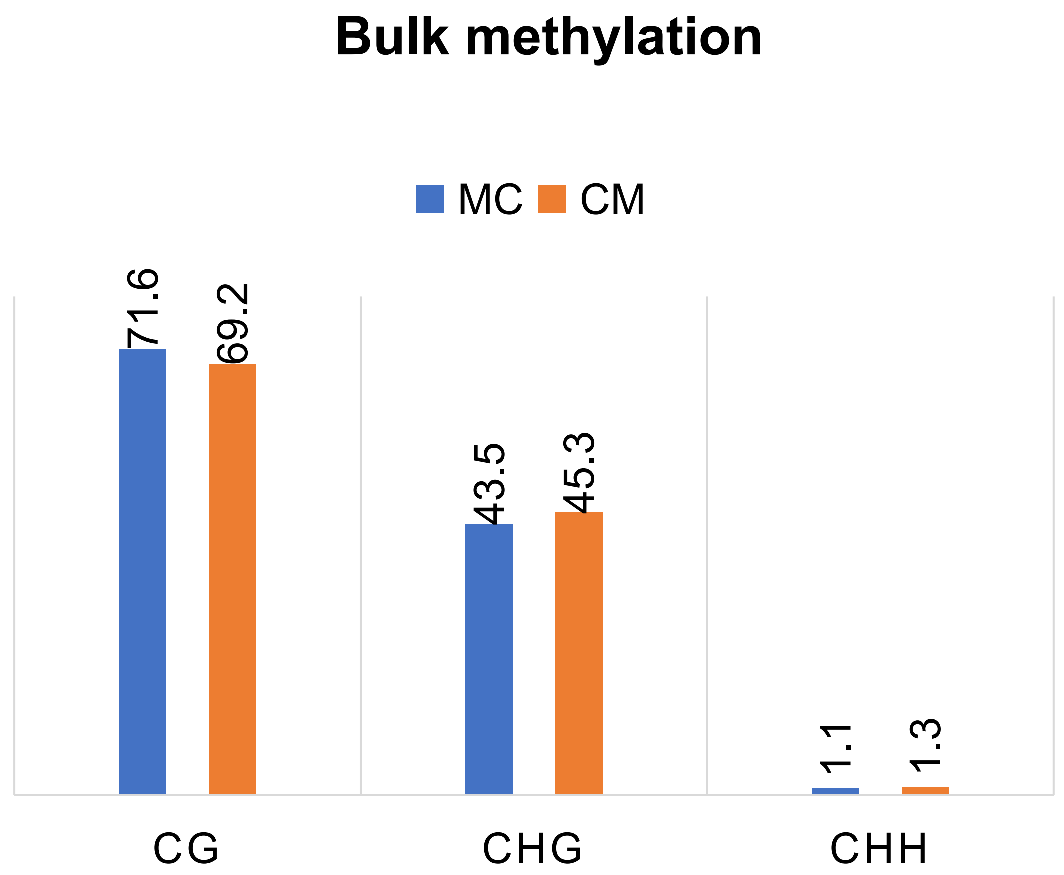

Fig. S3. The bulk DNA methylation levels in the endosperm of the CM and MC crosses. The y axis shows the bulk methylation in CG, CHG and CHH contexts, which are calculated by the ratio of the Cs with all the Cs and Ts from all the CG, CHG or CHH sites.

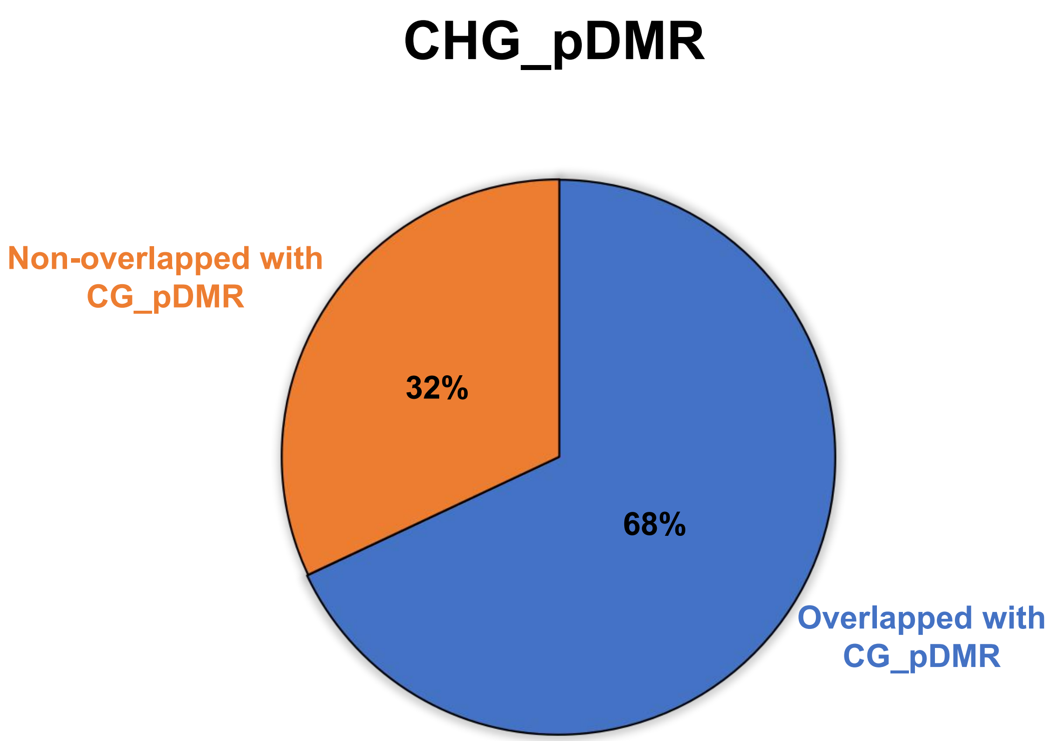

Fig. S4. The overlap between CG_pDMR and CHG_pDMR in MC/CM endosperm.

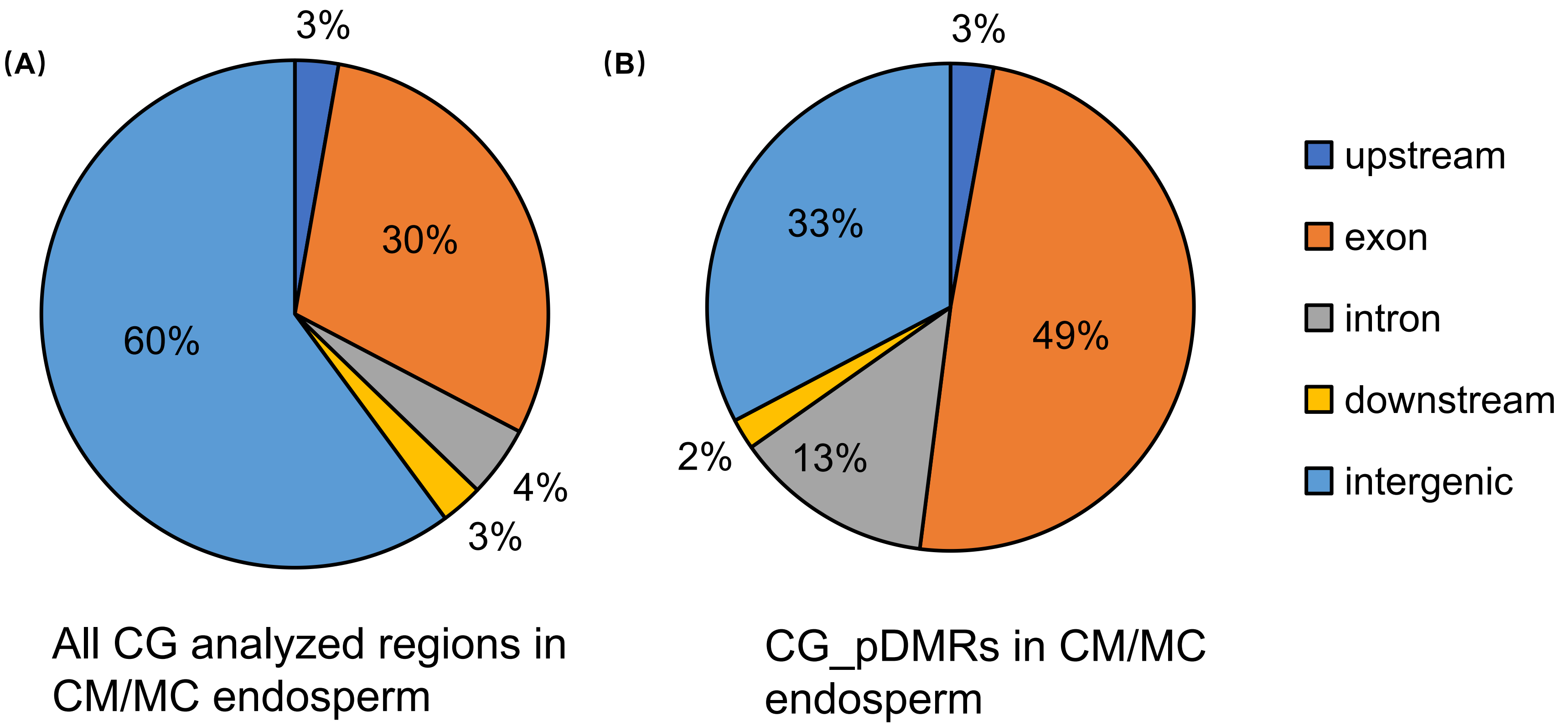

Fig. S5. The genomic distributions of CG_pDMRs.

The identified 1,367 CG_pDMRs were used to investigate the genomic distribution. All the analyzable regions in CG context were used as the control. The length of the extended upstream and downstream region is 2 kb.

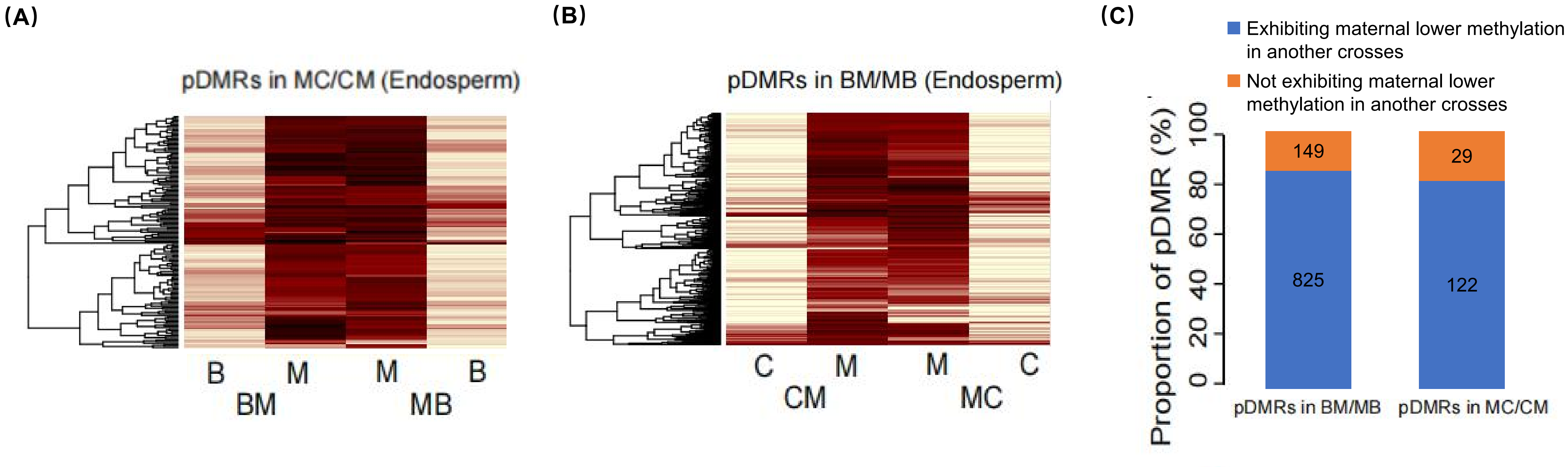

Fig. S6. Comparison of allele methylation in the CHG_pDMR regions identified in MC/CM and BM/MB endosperm.

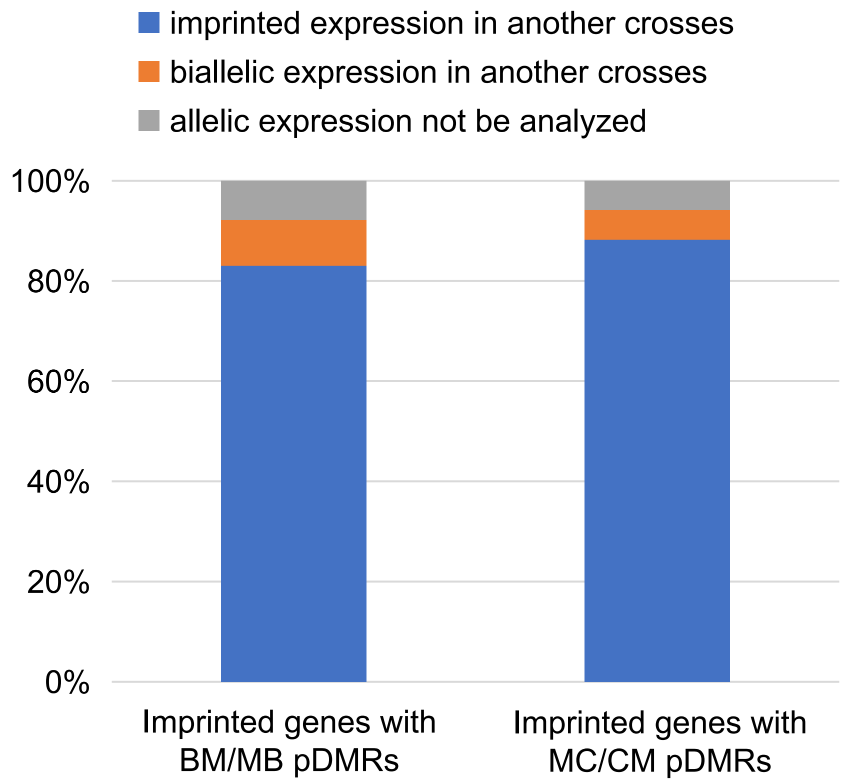

Fig. S7. The overlap between conserved pDMRs and imprinted genes in MC/CM and BM/MB endosperm.

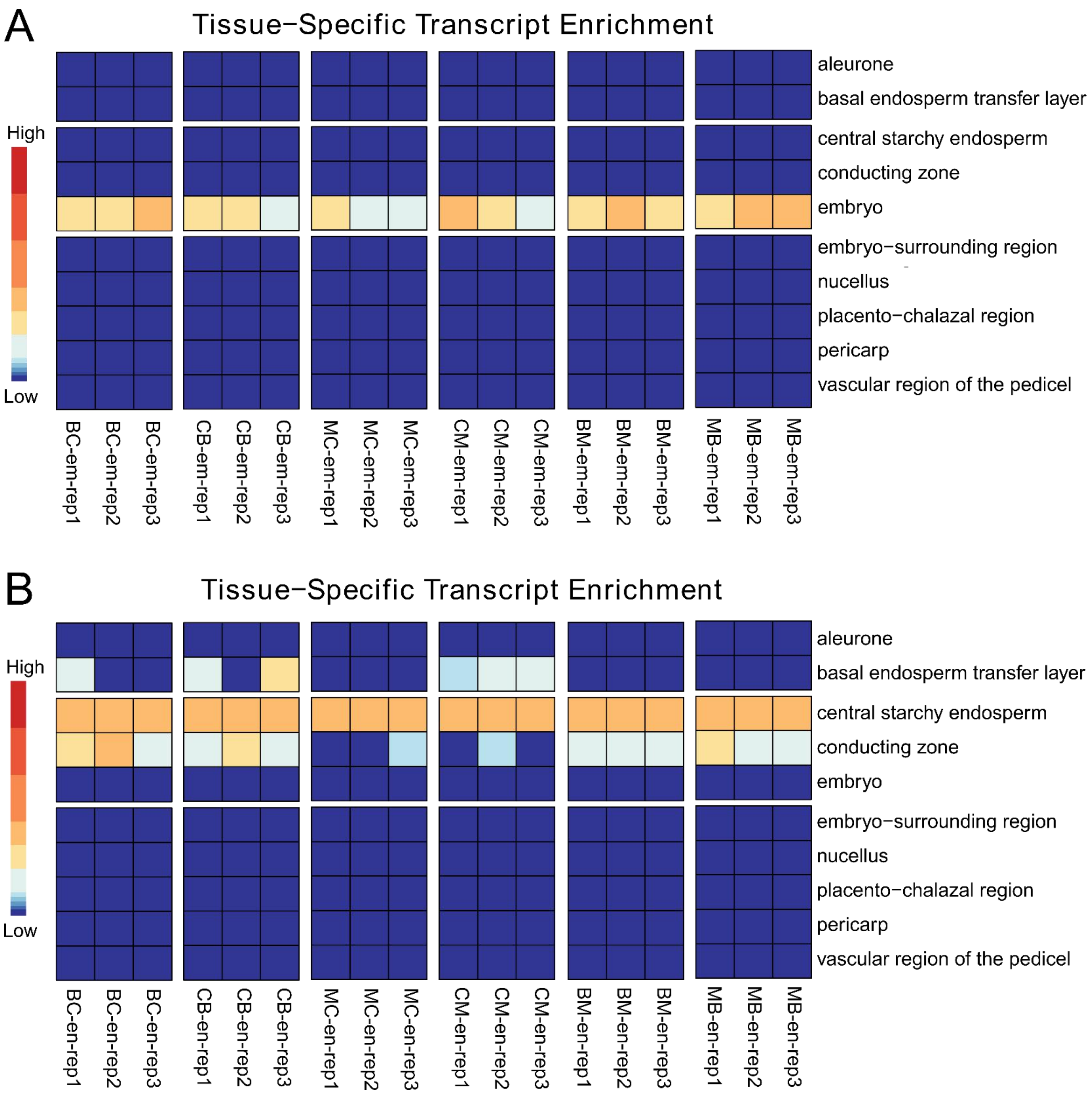

Fig. S8. Heat map illustrating results from the tissue enrichment test on embryo (A) and endosperm (B) transcriptomes from three reciprocal crosses. Rows represent tissue subregions including the aleurone (AL), the basal endosperm transfer layer (BETL), the central starchy endosperm (CSE), the conducting zone (CZ), the embryo (EMB), the embryo-surrounding region (ESR), nucellus (NU), placento-chalazal region (PC), pericarp (PE), and the vascular region of the pedicel (PED). as abbreviated in (A). Colors indicate the level of tissue enrichment according to the key.
